# Overview of recent advances in liposomal nanoparticle-based cancer immunotherapy

**DOI:** 10.1038/s41401-019-0281-1

**Published:** 2019-08-01

**Authors:** Ang Gao, Xian-li Hu, Madiha Saeed, Bin-fan Chen, Ya-ping Li, Hai-jun Yu

**Affiliations:** 1 School of Pharmaceutical Science, Jiangsu University, Zhenjiang, 212013 China; 2 State Key Laboratory of Drug Research & Center of Pharmaceutics, Shanghai Institute of Materia Medica, Chinese Academy of Sciences, Shanghai, 201203 China; 3 School of Chemistry and Molecular Engineering, East China Normal University, Shanghai, 200241 China; 4 University of Chinese Academy of Sciences, Beijing, 100049 China

**Keywords:** immunotherapy, liposomal NPs, dendritic cells, T cells, tumor cells, natural killer cells, macrophages, nanomedicine

## Abstract

The clinical performance of conventional cancer therapy approaches (surgery, radiotherapy, and chemotherapy) has been challenged by tumor metastasis and recurrence that is mainly responsible for cancer-caused mortalities. The cancer immunotherapy is being emerged nowadays as a promising therapeutic modality in order to achieve a highly efficient therapeutic performance while circumventing tumor metastasis and relapse. Liposomal nanoparticles (NPs) may serve as an ideal platform for systemic delivery of the immune modulators. In this review, we summarize the cutting-edge progresses in liposomal NPs for cancer immunotherapy, with focus on dendritic cells, T cells, tumor cells, natural killer cells, and macrophages. The review highlights the major challenges and provides a perspective regarding the clinical translation of liposomal nanoparticle-based immunotherapy.

## Introduction

Conventional cancer therapeutic strategies, including surgery, radiotherapy, and chemotherapy, have been applied in the clinic for several decades. A majority of tumors may be eliminated by applying these methods; however, metastasis and subsequent recurrences remain a formidable challenge in the clinic [1, 2]. Chemotherapy has long served as one of the main clinical treatment methods. However, multidrug resistance and off-target toxicity are questionable and the main limitations of chemotherapy [3]. Therefore, it is imperative to apply alternative methods, such as immunotherapy, that eradicate the primary tumor and tumor metastases as well as prevent recurrence by inducing immunological memory.

Combination therapy methods are of paramount importance in the clinic. Except for surgery and radiotherapy, other available methods require systemic administration to deliver cargo to tumor cells. Therefore, weak tumor targeting, severe side effects [4], rapid clearance from the blood, and drug resistance are some of the main limitations [5]. To overcome these shortcomings, various intelligent delivery platforms have been developed that may enhance tumor-targeting capabilities to achieve precise therapeutic performance [6]. The most popular delivery platform is nanoparticles (NPs), which can be divided into three types: micelles, liposomes, and inorganic NPs. For the sake of achieving controllability with micelles, polymers are designed to have different properties that ensure precise drug release at the tumor site, such as an acid-switchable micelle for photochemotherapy [7], a glutathione-sensitive prodrug micelle for chemoimmunotherapy [8] and an acid-activatable micelles for photoimmunotherapy [9]. However, the monolayer structure of micelles can only encapsulate specific hydrophobic drugs. Therefore, other drugs must be encapsulated by a covalent bond, which greatly limits drug release. Liposomes may be an alternative choice since both hydrophilic drugs and hydrophobic drugs can be synchronously delivered by liposomes due to the hydrophilic inner structure of liposomes [10], which greatly improves drug-loading efficiency. In addition, the cell membrane-like structure of liposomes provides efficient cell affinity and dramatically increases cellular uptake. Inorganic NPs have been widely used for cancer theranostic treatment in recent years because of their excellent physical and chemical properties; in particular, mesoporous silica, a typical inorganic material for NPs, has high drug-loading efficiency caused by surface micropores filling with drugs. However, inorganic materials cannot be metabolized; as a result, they can lead to serious tissue injury. Liposomes consist of lipids that act within the framework of the cell membrane, so they have lower organ toxicity and better biocompatibility than inorganic NPs. The positive surface charge of cationic liposomes enables them to achieve high-efficiency nucleic acid transfection. Moreover, the polyethylene glycol (PEG) shell ensures prolonged blood circulation in vivo [11].

**Fig. 1:**
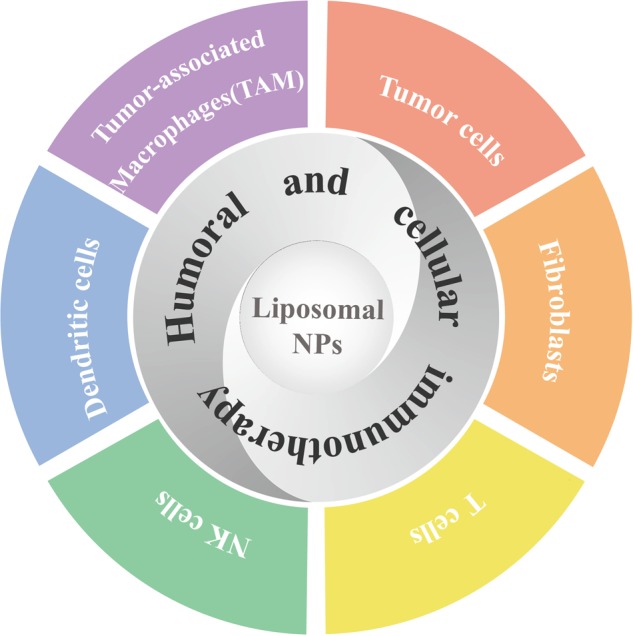
Schematic illustration of liposomal NPs used for cancer immunotherapy that target immune-related cells (e.g., NK cells, dendritic cells, tumor-associated macrophages (TAM), tumor cells, T cells, and fibroblasts) and activate the humoral or cellular immune response

Various phospholipid materials can achieve precise release in different environments, and multiple components facilitate their modification. Most immune agents are polypeptide, antibody, or nucleic acid drugs. Therefore, liposomes are considered ideal carriers for mediating effective cancer immunotherapy by activating either the humoral or cellular immune response [12] (Fig. 1).

As a systematic regulatory process, tumor immunity, including humoral and cellular immunity, can mobilize a variety of immune cells. There are three categories: messengers, killers, and regulators. Messengers are mainly antigen-presenting cells, specifically dendritic cells, macrophages, neutrophils, etc [13]. Cytotoxic T cells [14] and natural killer (NK) cells [15] are killers responsible for destroying tumor cells. Regulators, including regulatory T cells (Tregs), are involved in inhibiting the activity of APCs and the proliferation and differentiation of effector T cells [16]. This review provides a brief overview of the recent developments in liposomal NPs used for immunotherapy (Table 1).

**Table 1:** Preclinical studies of liposomal nanoparticle-based cancer immunotherapy

Immunotherapeutic modulators	Delivery platforms	Tumor model	Cell type	Refs
CpG and cisplatin	Liposomal NPs	Mouse model of melanoma	B16-F10	[62]
Mitoxantrone-treated tumor cells, anti-PD-1, and CpG	Hyaluronic acid-modified cationic lipid NPs	Mouse model of melanoma and colon carcinoma	B16-F10-OVA and CT26	[63]
TGF-β	Liposome-protamine-hyaluronic acid NPs	Mouse model of melanoma	B16-F10	[64]
IL-2 and IL-12	pDNA-loaded liposomal NPs	Mouse model of head and neck squamous cell carcinoma	SCC VII	[65]

## Impact of liposomal NPs on dendritic cells

Dendritic cells (DCs) are antigen-presenting cells (APCs) that act as a bridge between humoral and cellular immunity. The main roles of DCs are to capture, process, and present antigens to T cells. At the tumor site, DCs recognize and cross-present tumor antigens as designated peptides displayed by major histocompatibility complexes (MHCs). MHCs specifically interact with T cells via T-cell receptors (TCRs). This interaction elicits a series of T-cell-mediated immune responses and augments antitumor cytotoxicity [17]. However, only mature DCs can cross-present tumor antigens to T cells, and therefore, the first step is to induce DC maturation. An immature DC can recognize pathogens and antigens through pathogen recognition receptors (PRRs), such as Toll-like receptors (TLRs).

**Fig. 2:**
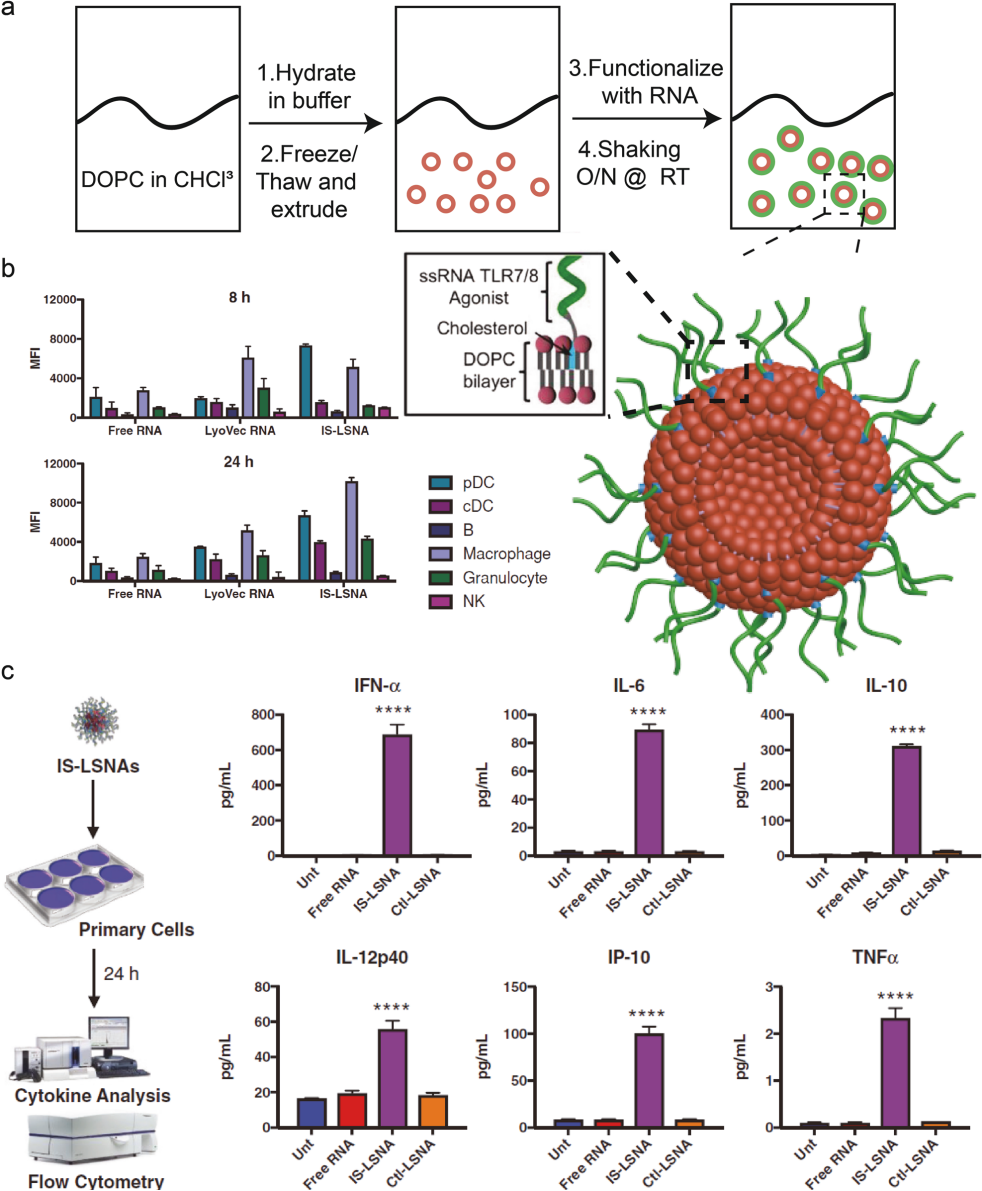
Fabrication of TLR7/8 agonist ssRNA-conjugated liposomal nanoparticles for DC-targeted cancer immunotherapy. **a** Chemical components and structure of immune-stimulatory spherical nucleic acids (IS-LSNAs). **b** Flow cytometric examination of IS-LSNA uptake in immune cells examined after 8 h or 24 h of incubation. The ssRNA-conjugated liposomal nanoparticles showed higher NF-κB activation efficacy than did free RNA or RNA delivered by a cationic lipid. **c** Cytokine secretion by primary immune cells after 24 h of incubation with IS-LSNA NPs. Adapted with permission from ref. [18]. ****P < 0.0001. Copyright (2018) John Wiley & Sons, Inc

Guan et al. developed immunostimulatory spherical nucleic acids (IS-LSNAs) that can selectively target TLRs 7/8. The IS-LSNAs were taken up by plasmacytoid dendritic cells and triggered an NF-κB pathway to activate DCs. The antigen-loaded core of the liposomes ensured enhanced antigen-specific T-cell priming (Fig. 2) [18]; TLR7/8 and TLR3 can also be exploited to induce DC maturation.

Varypataki et al. designed a cationic liposome with the TLR3 ligand poly(inosinic-polycytidylic acid) (poly(I: C)), which encapsulated a synthetic long peptide (SLP) harboring an antigen-specific cytotoxic T-cell (CTL) epitope (SIINFEKL). The SLP-loaded liposomal formulation efficiently delivered cargo to DCs in vitro and induced a functional CD8^+^ T-cell-mediated immune response in vivo [19, 20].

The targeted delivery of tumor-associated antigens (TAAs) to DCs has been frequently applied to enhance antitumor immune responses. However, the delivery of nucleic acids (NAs) encoding TAAs is an alternative method. Markov et al. designed a mannose receptor-targeted liposome to deliver plasmid DNA encoding EGFP and total tumor RNA to DCs. The mannosylated liposomes (ML) caused a five- to six-fold decrease in the number of melanoma lung metastases and enhanced the antitumor response in a murine melanoma model [21].

Liu et al. synthesized amphiphilic vaccines (amph-vaccines) consisting of an antigen that was linked to a lipophilic albumin-binding tail, while the lipid head was bound to single-stranded oligonucleotides containing unmethylated cytosine-guanine motifs (CpG DNAs) that were capable of interacting with TLR9. The amph-vaccine codelivery strategy efficiently transported peptide antigens and CpG DNAs, while efficient lymph node targeting enhanced T-cell responses and antitumor efficacy [22].

In addition to acting as carriers, liposomes are capable of activating a potent immune response. For example, using lipids from archaea, i.e., archaeosomes, has been presented as a promising approach to activate DCs and provide adjuvant stimulation to the immune response [23]. Archaeosomes provide an effective platform for antigen delivery and thus induce humoral and cellular immunity; however, they are correlated with increased expression of programmed death protein-1 (PD-1). The combination of archaeosome-based vaccines with checkpoint inhibition, such as anti-PD-1 or anti-CTLA-4 therapy, may improve the strength of T-cell responses and long-term protection [24]. Therefore, lipids from archaea may be an attractive adjuvant in vaccine formulations.

## Impact of liposomal NPs on tumor cells

Tumor eradication and recurrence suppression are the ultimate goals of cancer immunotherapy. A straightforward strategy is to deliver an immunogen to tumor cells that results in the activation of immunogenic cell death [25].

Immunogenic cell death (ICD) is associated with a constellation of alterations in cell composition and ultimately the release of signals that activate the maturation of DCs, presentation of tumor antigens to T cells, and subsequent T-cell response. A range of cytostatic agents, such as anthracyclines, oxaliplatin, and bortezomib, as well as radiotherapy and PDT, can lead to ICD [26]. Endogenous danger-associated molecular patterns (DAMPs), including surface-exposed calreticulin (CRT), high mobility group protein B1 (HMGB1), heat shock proteins (HSPs), and secreted ATP, are considered the main factors of ICD and can elicit an adequate immune response. DAMPs are of paramount importance in anticancer therapy due to their interactions with immune systems. Calreticulin (CRT) can elicit an anticancer immune response by translocating to the surface of dying cells from the lumen of the endoplasmic reticulum, thus sending an “eat me” signal to professional phagocytes, including macrophages, neutrophils, and dendritic cells [27]. HSPs, such as HSP 70 and HSP 90, can also be translocated to interact with APC surface receptors, such as CD91 and CD40. The cross-presentation of tumor antigens on MHC-1 can facilitate CD8^+^ T-cell-mediated immune responses. Amphoterin (HMGB1), a late apoptotic marker, is released into the extracellular space and binds with several PRRs, such as TLR2 and TLR4 on APCs. ATP is considered an “eat me” signal that recruits monocytes to the site of apoptosis [28].

Doxorubicin (DOX) is an anthracycline antitumor drug that is used to induce ICD. Ultrasound-controlled DOX-encapsulated liposomes can improve the accumulation of DOX in the tumor cell nucleus and ultimately enhance ICD induction [29]. However, ICD activation alone does not achieve significant results because there are many immunosuppressive pathways, such as the overexpression of indoleamine 2,3-dioxygenase (IDO-1) in the tumor microenvironment, which can upregulate the catabolism of tryptophan [30]. To improve therapeutic performance, combining ICD with other modalities is considered a good strategy. Therefore, Lu et al. designed a chemoimmunotherapy approach to codeliver DOX and IDO-1 inhibitor. Doxorubicin can effectively induce ICD, while indoximod can interfere with immunosuppression; thus, binary immunotherapy significantly enhances the antitumor immune response [31].

Programmed death ligand-1 (PD-L1)/PD-1 pathways also play major roles in tumor immunosuppression. Following the release of interferon gamma (IFN-γ), tumor cells express PD-L1, which binds with PD-1-expressing T cells, and thus IFN-γ renders T cells inactive and allows tumors to evade the immune response. Therefore, much attention is paid to blocking these PD-L1/PD-1 interactions by using inhibitors and antibodies. Gu et al. designed pH-sensitive PD-LI-targeting docetaxel-encapsulated liposomes that can precisely target tumors. Due to their pH sensitivity, the liposomes could specifically release docetaxel after accumulating in tumor cells and indirectly activate the immune system by producing TAAs. Targeted checkpoint blockade relieved immunosuppression and improved the immune response in order to kill tumor cells [32–37]. Moreover, liposome-based dual-modality approaches involving photodynamic therapy (PDT) and photothermal therapy (PTT) have also been explored to improve immunological responses [38, 39].

**Fig. 3:**
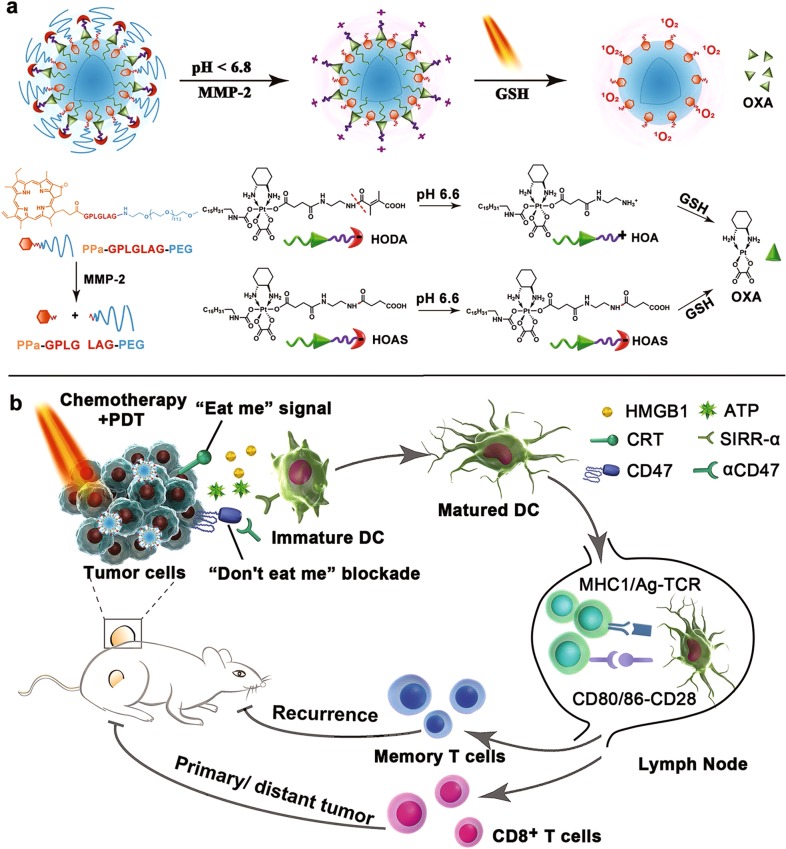
Schematic illustration of OXA prodrug-loaded liposomal vesicles for tumor cell-targeted combination cancer immunotherapy. **a** Chemical components of tumor microenvironment (TME)-activatable prodrug vesicles. **b** Mechanistic diagram of the prodrug vesicles for cancer immunotherapy. The OXA prodrug and photosensitizer delivered by the nanovesicles can use chemotherapeutic- and phototherapeutic mechanisms to trigger the ICD cascade in tumor cells. In addition, anti-CD47 antibody-mediated CD47 blockade can facilitate the phagocytosis of dying tumor cells by APCs to amplify antitumor immunity. Adapted with permission from ref. [40]. Copyright (2019) John Wiley & Sons, Inc

In addition to PD-L1/PD-1, CD47 is another immune checkpoint that can be targeted. CD47 is a transmembrane protein that sends a “don’t eat me” signal to DCs. Studies have revealed that CD47 blockade can promote DC maturation and T-cell priming. Zhou et al. designed a tumor microenvironment (TME)-activated prodrug liposome. This liposome consisted of a photosensitizer (PS) and oxaliplatin (OXA) codelivery platform. When the liposomal delivery system arrived at the tumor site, the matrix metalloproteinase-2 (MMP-2)-sensitive PEGylated PS cleaved the PEG shell at pH = 6.6. MMP-2-mediated cleavage of the PEG corona and exposure of the amine groups led to a surface charge reversal from negative to positive. The charge reversal promoted cellular uptake and prolonged tumor retention. The intracellular high glutathione concentration induced OXA release, while a 671-nm-wavelength laser ensured a photodynamic therapy (PDT) effect. Chemo-phototherapy triggered ICD, and the anti-CD47 antibody-mediated CD47 blockade relieved the immunosuppression (Fig. 3). This study suggests that combination therapy can promote DC maturation and antigen presentation, ultimately enhancing antitumor immunity. Moreover, such therapies can also induce an antitumor effect [40]. Combined PD-L1 and CD47 checkpoint blockade has also been reported to be an effective strategy. Liu et al. constructed a dual-targeting fusion protein that targeted both CD47 and PD-L1 using “knobs-into-holes” technology [41]. These studies imply that targeting innate and adaptive immune checkpoints (CD47 and PD-L1) can maximize the antitumor therapeutic effect and elicit more durable responses.

## Impact of liposomal NPs on T cells

CD8^+^ CTLs express the glycoprotein CD8 on the cell surface. CD8^+^ T cells recognize and kill tumor cells and can also secrete cytokines, such as IFN-γ, to stimulate tumor infiltration. Helper T cells are also known as CD4^+^ T cells and express the glycoprotein CD4 on their surface. Helper T cells mainly assist other T cells in immunological processes, including the activation of cytotoxic T cells [42]. Korsholm et al. described a cationic adjuvant formulation that can be formulated with different antigens and thus induce antigen-specific CD8^+^ T cells and effector-memory CD4^+^ T cells [43]. Agonists of the tumor necrosis factor (TNF) receptor family can stimulate an intense cancer-killing response. For example, CD137, which is expressed by activated T cells, can enhance the proliferation and cytotoxic activity of T cells once cross-linked [44]. Agonistic anti-CD137 antibodies and immunostimulatory cytokines, such as IL-2, can induce strong antitumor immunity. However, these agents may exhibit serious systemic toxicities, which impede their clinical use. Zhang et al. designed an immune agonist-anchoring liposome to deliver IL-2 and an anti-CD137 antibody. The rapid accumulation of the liposomes in tumors stimulated tumor infiltration by CD8^+^ T cells and cytokine and granzyme secretion and elicited effective antitumor activity while avoiding systemic toxicity [45].

**Fig. 4:**
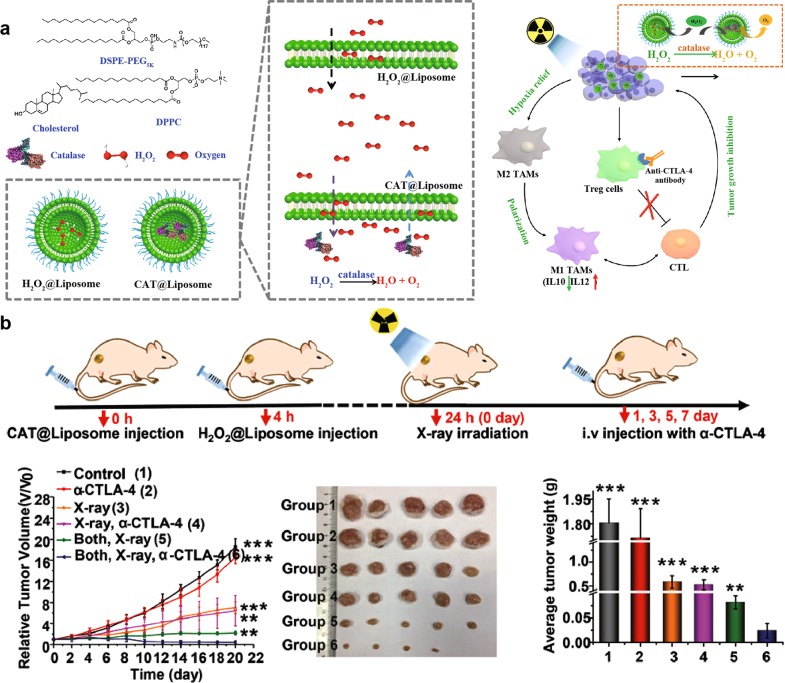
Schematic illustration of the structure and mechanism of CAT/H_2_O_2_@Liposomes for tumor cell and T-cell dual-targeted combination immunotherapy. **a** Catalase and H_2_O_2_ are encapsulated in liposomes containing DPPC, DSPE-mPEG_5k_, and cholesterol. Catalase can catalyze the decomposition of H_2_O_2_ into O_2_ by individual transportation of Catalase and H_2_O_2_ into tumors. The provided O_2_ relieves hypoxia and improves the ratio of M1/M2 tumor-associated macrophages. M1 macrophages can promote the activity of CTLs through the secretion of IL-12, and then an anti-CTLA-4 antibody can be used to reverse the transformation of CTLs into Tregs. **b** A combination therapy using radio- and immunotherapy can reduce the growth of tumors and even eliminate tumors. Adapted with permission from ref. [49]. **P < 0.01, ***P < 0.001 vs Both, X-ray, α-CTLA-4 (6). Copyright (2018) American Chemical Society

Tregs are suppressive T cells that can reduce the proliferation of effector T cells. The mechanism underlying Treg inhibitory functions is correlated with cytotoxic T lymphocyte-associated antigen 4 (CTLA-4), which is expressed on regulatory T cells. CTLA-4 can bind with CD80 or CD86, turn off the activity of APCs, and reduce T-cell priming [46, 47]. A PEGylated liposome with an encapsulated anti-CTLA-4 antibody can effectively deliver CTLA-4 to the tumor site and enhance the immune response [48]. An anti-CTLA-4 antibody plus radiotherapy approach can lead to a highly potent immune response. Song et al. designed a liposomal delivery system to deliver catalase and H_2_O_2_ to reverse hypoxia in the tumor site and to produce the enhancing immunological effect of anti-CTLA-4 therapy to enhance radio-immunotherapy (Fig. 4) [49].

## Impact of liposomal NPs on NK cells

Natural killer (NK) cells are critical cytotoxic lymphocytes in humoral immunity. Unlike T cells that need to bind with an MHC molecule to trigger tumor killing, NK cells can efficiently recognize stressed cells without the presence of MHCs or antibodies. The unique capacity of NK cells allows a relatively fast immune response. The function of NK cells is quite important because tumor cells missing MHCs cannot be recognized and killed by T cells [50].

Many malignant tumor cells can escape from T cells by downregulating the expression of MHC-1. Under these circumstances, NK cells play a vital role in the elimination of the tumor. Nakamura et al. designed YSK05-liposomes to encapsulate c-di-GMP for delivery to APCs. C-di-GMP could trigger APCs to secrete type 1 interferon (IFN) and inflammatory cytokines via the STING pathway and thus induce the activation of NK cells. NK cells in the tumor microenvironment could kill tumor cells [51].

Many studies have shown that overexpressed tumor necrosis factor-α-related apoptosis-inducing ligand (TRAIL) on the NK cell surface can interact with death receptors on tumor cells and induce cell apoptosis. Chandrasekaran et al. produced TRAIL liposomes, which were functionalized with an anti-NK1.1 antibody to target NK cells in tumor-draining lymph nodes. The TRAIL liposomes exhibited increased uptake by NK cells, improved delivery efficiency, and low systemic toxicity. This agent could efficiently eliminate metastatic tumor cells in tumor-draining lymph nodes due to its sustained retention [52].

NK-92 cells, as activated NK cells, can recognize tumor cells, home to tumor cells via activated receptors and release cytotoxic granules containing perforin and granzyme. The surface of engineered NK-92 cells can display a series of chimeric antigen receptors, such as anti-CD19 and anti-her2. The utilization of chimeric antigen receptors (CARs) as a drug delivery platform is a potent strategy to avoid off-target effects. Paclitaxel (PTX)-encapsulated liposomes bind to anti-CD19- or anti-Her-2-expressing NK-92 cells through conjugation to thiol on the cell surface. The PTX-liposome-CAR-NK cells can lead to an increase in IFN-γ^+^ cells and thus demonstrate precise tumor targeting and a powerful chemoimmunotherapy strategy [53]. The homing characteristic of NK-92 cells is mostly due to membrane proteins. Effective targeting can be achieved by exploiting all the proteins on the NK-92 cell membrane. Pitchaimani et al. designed an NK-92 cell fusogenic liposome encapsulating doxorubicin, DOX@NKsomes. The fused liposomes conserved all protein receptors on the NK cell membrane, such as CD56, NKG-2D, and NKp30. Therefore, they could recognize tumor cells via NK cell markers and effectively fuse with the tumor cells to release doxorubicin into the tumor cell cytoplasm [54].

## Impact of liposomal NPs on macrophages

In tumor immunotherapy, macrophages play an essential role due to their complex function in the immune system. Initially, macrophages can eliminate tumors by directly engulfing cancer cells that do not express healthy proteins on their surface [55]. The macrophages can then present TAAs after digestion. At the same time, macrophages act as secretory cells that can secrete cytokines to directly kill tumor cells, regulate the immune response and inflammation development, and indirectly inhibit tumor growth [56].

Tumor-associated macrophages (TAMs) are active immune cells in the solid tumor environment because they are closely related to the inflammation induced by the tumor [57]. In pancreatic cancer, many studies have confirmed that infiltrating macrophages mediate tumor progression in invasive tumors and early preinvasive pancreatic intraepithelial precursor lesions. Further studies have revealed that the depletion of TAMs by liposomal clodronate can dramatically reduce tumor metastasis [58, 59].

**Fig. 5:**
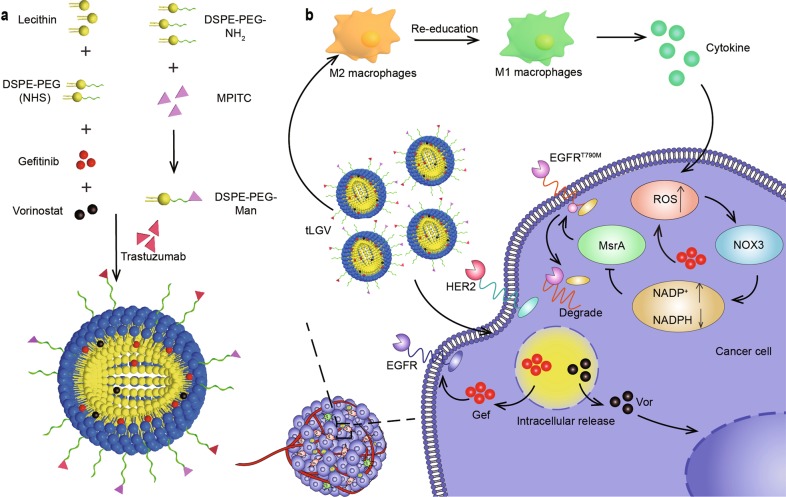
Schematic illustration of the codelivery liposome structure and immunotherapy mechanism. **a** Gefitinib and vorinostat were encapsulated in the lipid layer. Mannose/trastuzumab were decorated on DSPE-PEG_2k_ around the cell surface to target TAMs and tumor cells. **b** The dual-targeting liposome could repolarize tumor-promoting M2 TAMs into antitumor M1 TAMs, and the M1 TAMs could increase ROS levels in cancer cells, suppressing the expression of MsrA, which protect the T790M mutation in EGFR from oxidation. As a result, the macrophage-targeted liposomes could resensitize H1975 cells to gefitinib by means of EGFR^T790M^ degradation through the ROS/NOX3/MsrA axis. The dual-targeting liposome provides a new idea for tumor therapy via TAM-reprogramming strategies. Adapted with permission from ref. [60]. Copyright (2018) American Chemical Society

TAMs are divided into two types: M1 and M2. M1 macrophages are categorized as antitumor macrophages owing to their proinflammatory and cytotoxic functions, while M2 macrophages are considered protumor macrophages, due to their anti-inflammatory function. In clinical trials, patients who developed more M2 macrophages than M1 macrophages had a higher degree of tumor malignancy and worse overall survival, whereas patients who developed more M1 macrophages than M2 macrophages exhibited opposite effects. Exploiting this fact, a strategy of M2 macrophage re-education into M1 macrophages has been proposed for effective cancer therapy. Peng et al. designed a gefitinib/vorinostat codelivery liposome decorated with mannose/trastuzumab for dual targeting to transport the liposome to both tumor cells and macrophages. Gefitinib and vorinostat could downregulate M2-polarizing pathways and thus enhance the re-education of M2 macrophages. In addition, vorinostat in tumor cells could overcome resistance to gefitinib via deprotection of the T790 mutation (Fig. 5) [60]. In addition to molecular drugs, anti-CD163 antibodies can also achieve reprogramming of TAMs from the M2 phenotype into the M1 phenotype by inhibiting the expression of STAT3-regulated genes using corosolic acid (CA)-packaged, CD163-targeting, long-circulating liposomes [61].

## Summary and perspectives

In summary, tumor immunotherapy can be classified as exploiting humoral and cellular immunity. In humoral immunity, NK cells directly recognize and kill tumor cells. In cellular immunity, the process can be divided into three steps: immune activation, direct enhancement of the activity of effector T cells, and relief of  immune suppression.

There are two main methods to activate an immune response. One is to directly deliver tumor-associated antigens or nucleic acids and DC cell agonists, such as TLR agonists, to promote DC maturation, antigen cross-presentation, and T-cell priming. The other is to induce ICD in tumor cells by delivering anthracycline chemotherapeutic drugs or photodynamic therapy/radiotherapy, activating immunity through the ICD mechanism. Both mechanisms promote T-cell proliferation, differentiation into CTLs and secretion of IFN-γ, TNF-β, and other cytokines.

To improve effector T-cell activity, the delivery of cytokines and costimulatory molecules can enhance T-cell proliferation and cytotoxicity and thereby effectively induce antitumor functions. Regarding immunosuppression relief, the general strategy is to use immune checkpoint blockade, including the blockade of PD-1/PDL-1, CD47, and CTLA-4. The blockade of immune checkpoint pathways achieved by delivering blocking antibodies or small molecule inhibitors can mitigate the “don’t eat me” signal and enhance the tumor-killing ability of T cells. Other approaches are to adjust the biochemical environment of immunosuppression in tumor tissue, reverse the polarization of M2 macrophages by supplying oxygen to tumor tissue, reduce the degradation of tryptophan in tumor tissue and tumor-draining lymph nodes by inhibiting the activity of IDO, reduce the metabolism of tryptophan, restore the normal proliferation and differentiation of activated T cells, and inhibit the production of regulatory cells. As a conventional delivery platform, liposomes show notable superiority in cancer immunotherapy. However, the targeting efficiency of liposomal NPs is still less than satisfactory due to the presence of the same ligands on different cells; for example, PD-L1 can be overexpressed by DCs and TAMs as well as some tumor cells. Future research should explore the unique ligands (neoantigens) of tumor cells to achieve improved clinical performance. We believe that liposomal NPs will be used for safe and high-performance clinical applications in cancer immunotherapy.
